# Induction and Role of Indoleamine 2,3 Dioxygenase in Mouse Models of Influenza A Virus Infection

**DOI:** 10.1371/journal.pone.0066546

**Published:** 2013-06-13

**Authors:** Lei Huang, Lingqian Li, Kim D. Klonowski, S. Mark Tompkins, Ralph A. Tripp, Andrew L. Mellor

**Affiliations:** 1 Cancer Immunology, Inflammation and Tolerance Program, Cancer Center, Georgia Regents University, Augusta, Georgia, United States of America; 2 Department of Radiology, Georgia Regents University, Augusta, Georgia, United States of America; 3 Department of Cell Biology, College of Arts and Sciences, University of Georgia, Athens, Georgia, United States of America; 4 Department of Infectious Diseases, College of Veterinary Medicine, University of Georgia Athens, Georgia, United States of America; 5 Department of Medicine, Georgia Regents University, Augusta, Georgia, United States of America; Federal University of São Paulo, Brazil

## Abstract

Influenza infection stimulates protective host immune responses but paradoxically enhances lung indoleamine 2,3 dioxygenase (IDO) activity, an enzyme that suppresses helper/effector T cells and activates Foxp3-lineage regulatory CD4 T cells (Tregs). Influenza A/PR/8/34 (PR8) infection stimulated rapid elevation of IDO activity in lungs and lung-draining mediastinal lymph nodes (msLNs). Mice lacking intact IDO1 genes (IDO1-KO mice) exhibited significantly lower morbidity after sub-lethal PR8 infection, and genetic or pharmacologic IDO ablation led to much faster recovery after virus clearance. More robust influenza-specific effector CD8 T cell responses manifested in lungs of PR8-infected IDO1-KO mice, though virus clearance rates were unaffected by IDO ablation. Similar outcomes manifested in mice infected with a less virulent influenza A strain (X31). IDO induction in X31-infected lungs was dependent on IFN type II (IFNγ) signaling and was restricted to non-hematopoietic cells, while redundant IFN type 1 or type II signaling induced IDO exclusively in hematopoietic cells from msLNs. Memory T cells generated in X31-primed IDO1-KO mice protected mice from subsequent challenge with lethal doses of PR8 (100×LD_50_). However recall T cell responses were less robust in lung interstitial tissues, and classic dominance of TCR Vβ8.3 chain usage amongst memory CD8^+^ T cells specific for influenza nucleoprotein (NP_366_) did not manifest in IDO1-KO mice. Thus, influenza induced IDO activity in lungs enhanced morbidity, slowed recovery, restrained effector T cell responses in lungs and shaped memory T cell repertoire generation, but did not attenuate virus clearance during primary influenza A infection.

## Introduction

Cells expressing IDO suppress innate and adaptive immunity in a range of clinically relevant syndromes including autoimmune, allergic and infectious diseases, cancer, pregnancy, and transplantation [1]. IDO expressed at local sites of chronic inflammation caused by tumors and some persistent infections suppresses natural and vaccine-induced immunity to allow these agents of disease to persist in immunocompetent individuals, despite the antigenicity of tumor cells and infected cells [2], [3], [4]. In 1979 Yoshida and colleagues reported that influenza A virus (PR8) infection up-regulated IDO enzyme activity rapidly in mouse lung homogenates, peaking at ∼120-fold over basal levels in uninfected lungs when viral burdens were decreasing due to host adaptive immunity [5]. The role of IDO during primary host responses to influenza infection has not been defined, but elevated IDO activity due to prior influenza infection interfered with host control of subsequent *Streptococcus pneumoniae* lung infections as treatment with the reversible IDO inhibitor 1-methyl-tryptophan (1MT) led to significant reduction in bacterial outgrowth in lungs and to reduced levels of IL-10 and TNFα [6]. Thus, IDO induced by primary influenza infection impedes host control of secondary bacterial lung infections, a major cause of patient mortality after influenza infection [7].

Tissue inflammation elevates IDO activity because IFNs (IFNαβ, IFNγ) stimulate IDO gene transcription, though poorly defined post-translational processes are also essential for IDO enzyme activity. Hematopoietic myeloid cells and non-hematopoietic (stromal) cells can express IDO at inflammatory lesions. IDO activity induced in inflamed lymph nodes (LNs) draining sites of tumor growth, and following topical exposure to a tumor promoter (phorbol ester) conferred dominant IDO-dependent regulatory phenotypes on DCs in dLNs [8], [9]. Splenic CD19^+^ DCs also expressed IDO and mediated IDO-dependent T cell suppression when mice were treated with soluble CTLA4 (CTLA4-Ig), relatively high doses of TLR9 ligands (CpG oligonucleotides) and DNA nanoparticles delivered systemically [10], [11], [12], [13], [14], [15]. In these and other settings of inflammation, induced IDO activity in DCs caused naïve CD4 T cells to convert into Foxp3-lineage regulatory T cells (Tregs) and stimulated pre-formed, resting Tregs to acquire potent regulatory phenotypes that attenuated effector T cell responses [16], [17], [18], [19]. Tumor cells and some stromal cell types such as epithelial cells and fibroblasts can also express IDO at sites of inflammation, and these cells may also promote local T cell regulation.

In the current study we investigated the role and mechanism of IDO induction in two murine models of influenza A infection using strain PR8 and the less virulent strain X31 by monitoring IDO induction in lungs and draining LNs and evaluating the consequences of ablating IDO1 genes, one of two homologous genes (IDO1 and IDO2) that encode IDO [20], [21], on the course of host primary and recall T cell responses to influenza infection.

## Materials and Methods

### Virus

Stocks of HKx31 (H3N2) (X31) and A/PR/8/34 (PR8) influenza virus were propagated in embryonated chicken eggs. Titers for X31 virus and PR8 virus were 1.5×10^7^ PFU/ml and 1.4×10^7^ PFU/ml respectively. Virus titers in tissues from infected mice were measured as 50% tissue culture infection dose (TCID_50_) using MDCK cells as described [22]. 50% lethal dose (mLD_50_) of PR8 virus was estimated using 6 to 8 week old WT female mice; 30% weight drop was used as the experimental endpoint.

### Mice and infections

All animal procedures were approved by the GRU animal usage committee (IACUC, AUP#2009-0052). C57BL/6 (B6) mice were purchased from Jackson Lab (Bar Harbor, Maine) or the National Cancer Institute (Frederick, MD) or bred at GRU. All genetically engineered mice were crossed onto the B6 background for at least 10 generations. IDO1-KO mice were described previously [23]; IFNγR1-KO mice were purchased (Jax Labs); IFNAR-KO mice were a kind gift from Dr. Dimitrious Moskophidis [24]. Mice were infected with influenza virus diluted in PBS with 0.1% endotoxin-free, Ig-free BSA (Gemini Bio-products, Calabasas, California) via intranasal inoculation. Mice were anesthetized with isofluorane prior to inoculation. A dose of 30% of mLD_50_ PR8 virus was used for sub-lethal primary infection and a dose of 100×mLD_50_ PR8 virus was used to challenge X31 primed mice to test for protection against lethal infection afforded by memory T cells. For infection induced morbidity experiments, WT and IDO1-KO mice with BALB/c or B6 genetic backgrounds (fully backcrossed) were infected with PR8 virus (30% of mLD50) and mice were weighed daily Comparable outcomes were obtained for mice with both genetic backgrounds (data not shown). To generate bone marrow chimeric mice, IDO1-KO mice were lethally irradiated (900 rad), injected (i/v) with 1×10^7^ nucleated bone marrow cells by tail vein injection. Recipient mice were allowed to reconstitute for >6 weeks before infection.

### Antibodies, peptides and tetramers

Fluorophore conjugate anti-mouse CD4 (clone GK1.5 or RM4-5), CD8 (clone 53.67), Ly6G (clone 1A8), Ly6C (clone HK1.4), IFNγ (clone XMG1.2), were obtained from BD Bioscience (San Jose, CA), eBioscience (San Diego, CA) or Biolegend (San Diego, CA). Mouse anti-human and mouse anti-IDO1 monoclonal antibodies (E7) were from Santa Cruz Biotechnology, and rat anti-mouse IDO1 monoclonal antibody (mIDO48) was from Biolegend. Mouse anti-influenza NP (ATCC clone H16-L104R5) antibody was purified from culture supernatant and biotinylated using NHS-biotin according to manufacturer's instructions (Pierce/Thermo, Rockford, IL). A mouse anti-TCR Vβ mAb screening panel (BD Biosciences, CA) was used to type TCR Vβ chains in NP_366_-specific T cells.

Peptides specific to H-2D^b^ restricted NP_366_, H-2D^b^ restricted PA, H-2K^b^ restricted PB1 and H-2IA^b^ restricted NP_311_ were synthesized at Biobasic (Toronto, Canada). MHC class I tetramers were prepared in house as described [24], [25]. MHC class I monomers were biotinylated using biotin ligase BirA (Avidity, Aurora, Co). Biotinylated monomers were coupled to PE-conjugated streptavidin (Invitrogen, Calsbad, CA) to form tetramers. MHC class II tetramers were acquired from the NIH/NIAID core facility. Cell-surface stainings for flow cytometry analysis were performed in PBS with 0.1% BSA except for MHC class II tetramers, when HBSS was used instead of PBS.

### Bronchoalveolar Lavage (BAL)

BAL samples were collected by inserting a catheter into a small opening in the trachea and then flushing the lung with PBS 3 times. BALs from individual mice were analyzed. Lung interstitial cells were extracted as described with minor modifications [26]. Briefly, mice were perfused with PBS (6 ml) via the right ventricle after BAL extraction. Lungs were digested in 400 U/ml type 4 collagenase (Worthington Biochemical, Lakewood, NJ), and cells released were centrifuged in 40% Percoll. For intracellular cytokine staining, cells were placed in 96 well plates and stimulated *ex vivo* with appropriate peptide plus brefeldin (3 µg/ml, eBioscience) and 5 U/ml hIL2 (Roche Bioscience, Palo Alto, CA) for 4 hours. For stimulation of CD8 T cells from BAL and lung, V-bottom plates were used to provide better cell contact. For stimulation of CD4 T cells from BAL and lung, V-bottom plates were used and 50,000 spleen cells from Thy1.1 mice were added as feeders. Cells were then surface stained with anti-mouse CD8 or CD4, fixed with Perm/Fix (BD bioscience) and stained to detect intracellular IFNγ and IL10. To detect total Th1 and Th17 cells T cells isolated from BAL and lung parenchyma were stimulated *ex vivo* with 100 ng/ml PMA+1.5 µM ionomycin plus brefeldin.

### Flow cytometry

Cells were analyzed on a FACS LSRII flow cytometer. DAPI was added prior to analysis tetramer stained samples to identify dead cells. Data were analyzed using FACS DIVA (BD Bioscience) or FlowJo (Tree Star, Ashland, OR) software.

### Immunohistochemistry and Western blotting

For immunohistological analysis, lungs were infused with 20% OCT diluted in PBS and snap frozen in isopentane/dry ice bath. Nonspecific staining was blocked using 2% BSA and endogenous biotin was blocked using a biotin/avidin blocking kit (Vectorlabs, Burlingame, CA). A mouse monoclonal anti-mouse/human IDO antibody (5 µg/ml, E7, Santa Cruz Biotechnology) was used on methanol fixed lung frozen sections to detect IDO using a M.O.M kit (Vectorlabs, Burlingame, CA). Biotinylated anti-NP antibody was used at a concentration of 10 µg/ml. For western blotting, protein concentrations in tissue homogenates were measured using Bradford reagent (Pierce/Thermo, Rockford, IL). IDO protein was detected using monoclonal rat anti-mouse IDO1 antibody (mIDO48) using a chemiluminescent reagent (Denville Scientific, South Plainfield, NJ).

### Tissue kynurenine and IDO activity

Snap frozen tissues were homogenized in PBS at concentrations of 100 mg/ml (lung) and 50 mg/ml (lymph nodes). Kynurenine (Kyn) and tryptophan (Trp) were measured by HPLC after de-proteination using a C18 reverse phase column [27]. Tissue Kyn was expressed as pmol/mg tissue and serves as an *in vivo* index of IDO enzyme activity. IDO enzyme activity in cell-free tissue homogenates (*in situ*) was measured as described [28]. IDO activity was calculated as pmol kynurenine/hr/mg tissue.

## Results

### Influenza PR8 infection induces IDO activity in lungs and mediastinal lymph nodes (msLNs)

**Figure 1:**
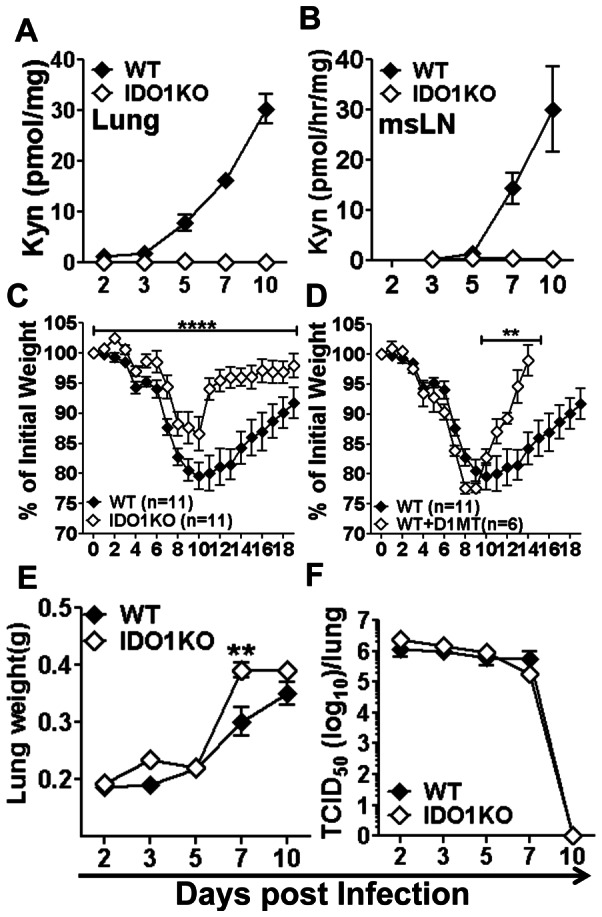
IDO increases morbidity and slows recovery in PR8-infected mice. B6 (WT) or IDO1-KO mice were infected with PR8 virus (50 PFU, i/n). **AB.** Tissue kynurenine (Kyn) in lung homogenates (A) and IDO enzyme activity in msLN homogenates (B) were estimated by HPLC. **CD.** Morbidity was assessed by monitoring weight loss after infection (normalized to initial weights) in BALB/c (WT), IDO1-KO (C) and BALB/c (WT) mice given oral D1MT during infection (D). **EF.** Lung weights (E) and lung virus loads (F) were also evaluated. Data are the means (+/−1sd) of >3 mice/group from at least two independent experiments unless otherwise stated in the figure. **p<0.01, ****p<0.0001.

To evaluate IDO enzyme activity during influenza infection B6 mice were infected with sub-lethal doses (50 PFU/mouse, i/n) of influenza A/PR/8/34 (PR8) virus and levels of the tryptophan catabolite kynurenine (Kyn) released by cells expressing functional IDO enzyme activity were assessed in lungs and msLNs by performing HPLC analysis on tissue homogenates. Kyn levels were elevated significantly in PR8-infected lungs, relative to basal levels in uninfected lungs, from 3–5 days post infection (dpi), and Kyn levels continued to increase until >10 dpi (Fig. 1A). Increased Kyn levels were also detected in msLNs starting slightly later (5–7 dpi) after infection (Fig. 1B). No kynurenine was detected in lungs or msLNs of IDO1-deficient (IDO1-KO) mice with B6 backgrounds (Fig. 1AB, open symbols), indicating that induced IDO enzyme activity is encoded by IDO1, not IDO2 [20], [21]. These data confirm and extend previous studies showing dramatic increases in lung IDO activity induced by PR8 infection [5].

### IDO enhances morbidity following PR8 infection

Sub-lethal infection of BALB/c mice with PR8 induced progressive reduction in body weight peaking at ∼20% weight loss at 11 dpi (relative to initial body weights) followed by gradual recovery (Fig. 1C, closed symbols). Sub-lethal PR8 infection caused significantly lower morbidities in BALB/c mice lacking intact IDO1 genes (IDO1-KO mice) as peak weight loss was ∼13%. Moreover, recovery from influenza began earlier and progressed faster in IDO1-KO mice (Fig. 1C, open symbols). Oral treatment (*ad libitum*) of BALB/c mice with the IDO-specific inhibitor 1-methyl-[D]-tryptophan (D1MT) during PR8 infection had no effect on morbidity induction but mice recovered weight much faster following viral clearance (Fig. 1D). At 7 dpi, when sterile PR8 clearance occurred in this model, lung weights were significantly higher (20–25%) in PR8-infected IDO1-KO mice relative to WT (BALB/c) controls (Fig. 1E), indicating that inflammatory infiltrates were more robust in IDO1-KO mice. However, no significant differences in lung viral burdens were detected during infection (Fig. 1F). Comparable disparities in PR8 induced morbidity and recovery rates after virus clearance were observed using WT and IDO1-KO with B6 backgrounds (data not shown), indicating that this phenomenon was not strain-specific. Thus, induced host IDO activity in PR8-infected lungs drives higher morbidity and slows recovery following primary influenza infection.

### IDO ablation enhances influenza-specific CD8 T cell responses to primary PR8 infection

**Figure 2:**
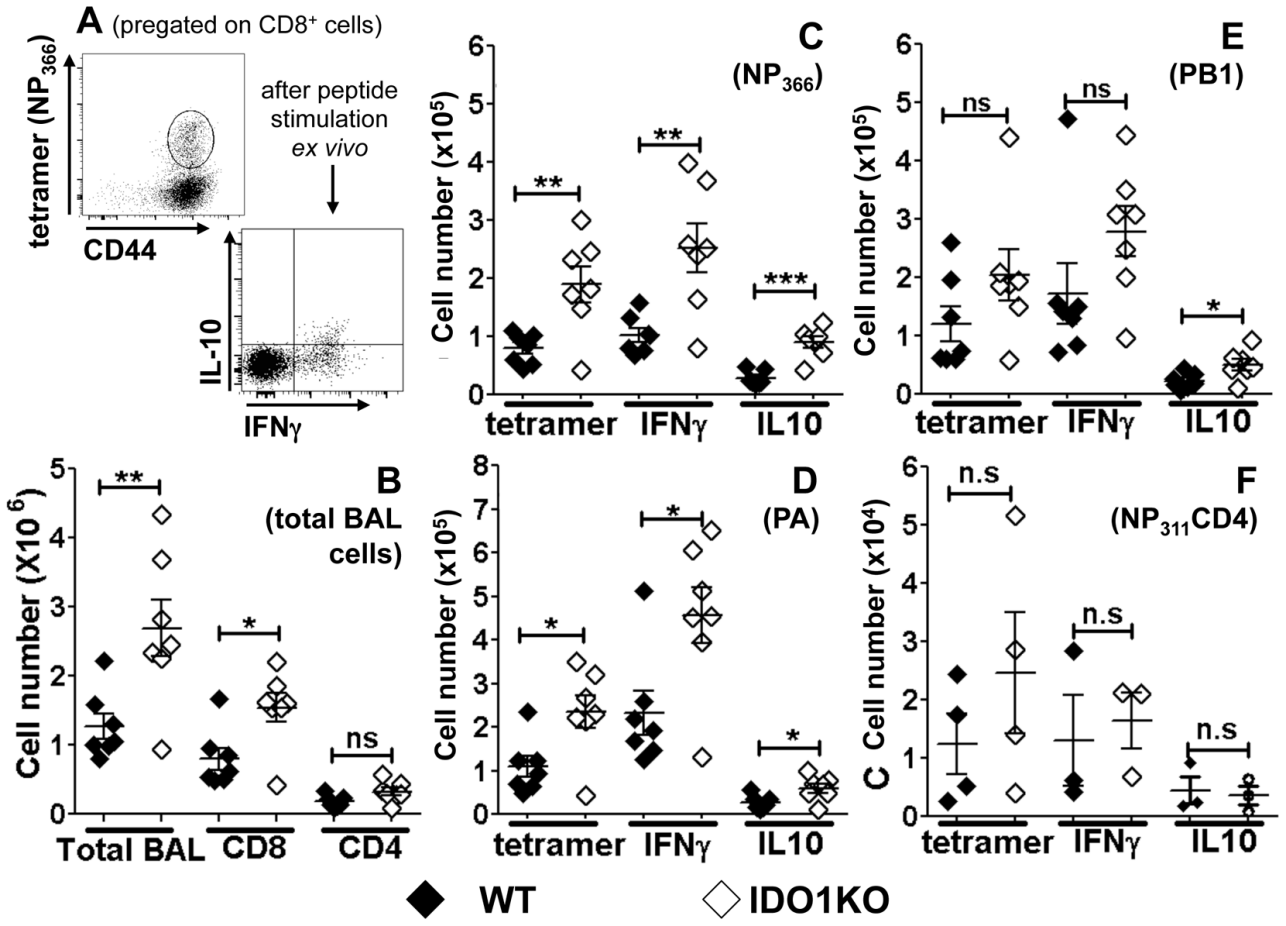
IDO ablation enhances influenza-specific CD8 T cell responses to PR8 infection. B6 (WT) or IDO1-KO mice with B6 backgrounds were infected with PR8 virus (50 PFU, i/n) at sub-lethal doses, and 10 days after infection bronchial alveolar lavage (BAL) cells and T cells were counted. **A.** Representative FACS gating and analysis strategy to detect NP_366_-specific CD8 T cells using tetramers (upper panel) and intracellular cytokine (IFNγ, IL-10) expression after peptide specific stimulation for 2 hrs *ex vivo* (lower panel). **B–F.** Numbers of total BAL (B) and NP_366_ (C), PA (D), PB1 (E) CD8 and NP_311_ (F) CD4 specific T cells. Data were analyzed using Student's unpaired *t* test; *p<0.05, **p<0.01, ***p<0.001, ns, not significant.

To assess if IDO1 gene ablation affected primary T cell responses to PR8 infection IDO1-KO mice with B6 backgrounds were infected with sub-lethal doses of PR8 (50 PFU/mouse, i/n) and virus-specific T cell responses were evaluated (at 10 dpi) by staining bronchial alveolar lavage (BAL) cells with anti-CD8 mAbs plus MHC class I tetramer reagents that bind to T cells specific for influenza nucleoprotein NP_366_, PA, or PB1 epitopes, and anti-CD4 mAbs plus MHC class II tetramer reagents specific to NP_311_ and analyzing cells by flow cytometry using the representative gating strategy shown for NP_366_-specific CD8 T cells (Fig. 2A). Numbers of cells and CD8^+^ T cells were significantly higher in BAL from PR8-infected IDO1-KO mice, relative to WT control mice (Fig. 2B). More influenza NP- and PA-specific BAL CD8^+^ T cells were present, and higher proportions of these cells expressed intracellular IFNγ and IL-10 in PR8-infected IDO1-KO mice (Fig. 2C and D). Small increases in PB1-specific CD8^+^ T cells (Fig. 2E), total CD4^+^ (Fig. 2B) and NP_311_-specific CD4^+^ T cells (Fig. 2F) were observed in BAL of PR8-infected IDO1-KO mice, but these increases were not statistically significant. Thus more robust influenza-specific effector CD8^+^ T cell (CTL) responses manifested in mice lacking intact IDO1 genes, indicating that influenza-induced IDO activity attenuates clonal expansion and/or inhibits access of CTLs into infected lungs of IDO-sufficient mice.

### Influenza infection stimulates increased IDO enzyme activity in lungs and msLNs

**Figure 3:**
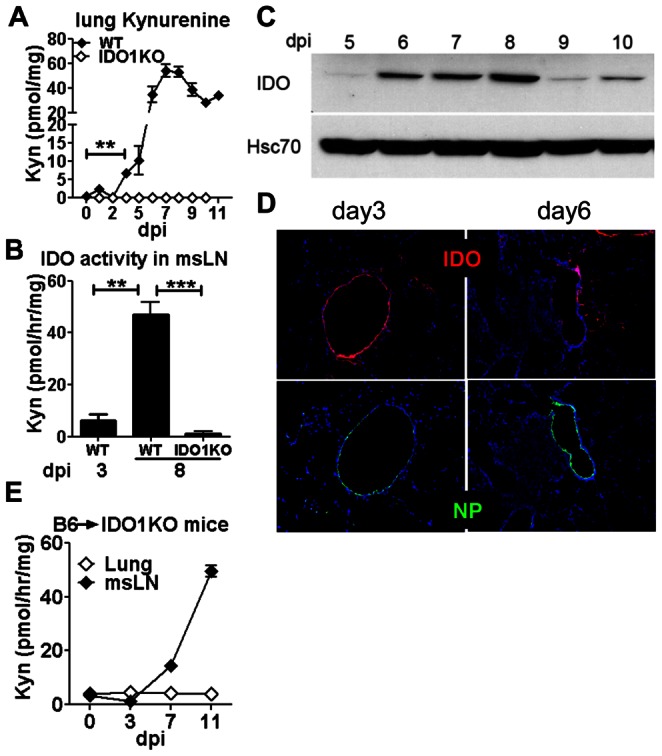
Influenza A infection stimulates IDO in lungs and draining msLNs. Mice were infected with 750 PFU of X31. IDO expression and activity in lungs and msLNs were measured at the times indicated after infection. **ABE.** Tissue kynurenine (A) or IDO enzyme activity (B, E) were assessed in lungs and msLNs of X31-infected WT (B6), IDO1-KO (B6) and B6→IDO1-KO chimeric mice. **CD.** IDO protein was assessed in lungs of X31-infected WT mice by Western blot (C) and immunohistochemical analyses (D). Influenza infected lung cells were identified by detecting NP in adjacent sections (D). Data are the means (+/−1sd) of >3 mice/group from at least two independent experiments. Original magnifications, 200×. **p<0.01, ***p<0.0001.

**Figure 4:**
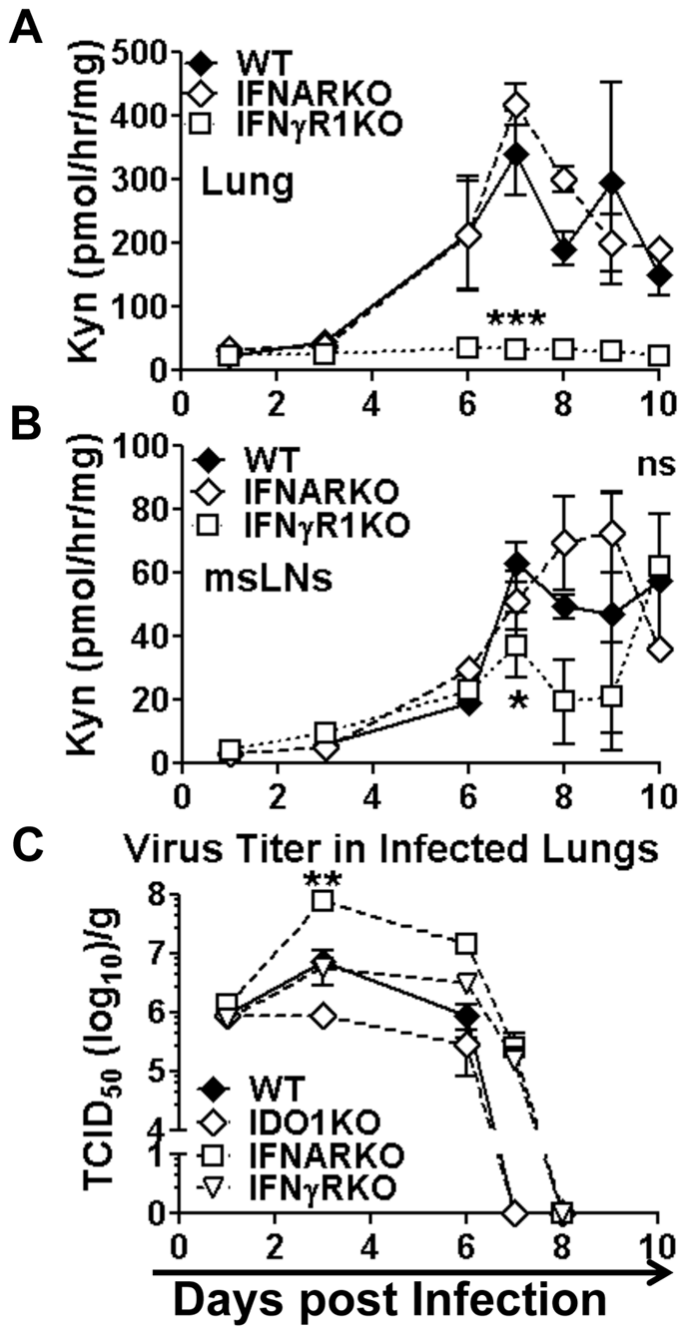
Interferon signaling requirements to induce IDO. **AB.** IDO enzyme activity was assessed in lungs (A) and msLNs (B) of WT (B6), IFNAR-KO and IFNγR1-KO (B6) mice after X31-infection. **C.** Virus titers in lungs of WT, IFNAR-KO and IFNγR1-KO mice. Statistical analyses were performed on selected time points using Student's unpaired *t* test. *p<0.05, **p<0.01, ***p<0.001, Data were compiled from two experiments with 2–6 mice/time point.

As sub-lethal PR8 infection caused substantial differences in morbidity between WT and IDO1-KO mice (Fig. 1), we used a less virulent strain (X31) to evaluate the role of IDO in host-pathogen interactions. B6 mice were infected with relatively low doses of X31 (750 PFU/mouse) that did not cause significant morbidity (data not shown), and Kyn levels were detected in lung homogenates from X31-infected mice. Similar to PR8-infected mice, Kyn levels in lungs from X31-infected WT mice increased from 3–6 dpi and peaked at 7–8 dpi (Fig. 3A). Kyn levels declined slowly after 8 dpi but did not return to basal levels by >11 dpi, even though sterile virus clearance occurred in lungs by 7 dpi (Fig. 4C). As Kyn is excreted rapidly, these data indicate that influenza-induced IDO activity remained abnormally high in lungs for a long time after virus was eliminated. As expected, lung Kyn levels were below detection limits during primary infection and after viral clearance in X31-infected IDO1-KO mice (Fig. 3A).

To evaluate if IDO activity was also induced in msLNs cell-free homogenates of msLNs were incubated with enzyme substrate cocktail for 2 hours and Kyn generated *ex vivo* was assessed by HPLC as described in Materials and Methods. Similar to Kyn levels in lungs, IDO activity in msLNs increased substantially between 3 dpi and 8 dpi, while no IDO activity was detected in msLNs of X31-infected IDO1-KO mice (Fig. 3B). Consistent with these findings, IDO protein expression was substantially higher in lungs of X31-infected mice from 6–10 days post-infection and remained higher than basal levels until >13 dpi (Fig. 3C, and data not shown). Immunohistologic analyses revealed induced IDO expression in the epithelial linings of a small number of bronchial alveolar vesicles as early as 3 dpi, but only at local sites where active influenza infection was also detected, as evidenced by expression of influenza nucleoprotein (NP) in adjacent tissue sections (Fig. 3D). Thus, IDO was induced rapidly in response to local influenza infection, and IDO activity encoded exclusively by IDO1 genes continued to increase during infection and remained substantially higher than basal levels in lungs and msLNs long after sterile virus clearance occurred. These data are consistent with findings reported by Yoshida and colleagues who measured lung IDO activity in ICR mice infected with PR8 [5], except that IDO activity peaked later at ∼11 dpi in this study.

### Influenza infection induces IDO in distinct cell types in lungs and msLNs

To evaluate if influenza infection induced IDO in hematopoietic or non-hematopoietic cell compartments we lethally irradiated IDO1-KO mice and reconstituted them with bone marrow from B6 (WT) donor mice to generate B6→IDO1-KO chimeric mice. After reconstitution (8 weeks) chimeric mice were infected with X31 and IDO enzyme activity was assessed in lungs and msLNs. IDO activity in lungs of infected B6→IDO1-KO chimeric mice remained at basal levels, relative to uninfected controls (Fig. 3E, open symbols). In contrast, IDO activity in msLNs from X31-infected B6→IDO1-KO chimeric mice increased between 3–11 dpi (Fig. 3E), and was comparable with increased IDO activity observed in X31-infected B6 (WT) mice (Fig. 3B). Thus, non-hematopoietic cells accounted for all lung IDO activity induced by X31 infection, while hematopoietic cells accounted for all the IDO activity induced in msLNs of X31-infected mice, indicating that distinct cell types expressed IDO activity in these tissues.

### Influenza induces IDO via distinct interferon signaling pathways in lungs and msLNs

To assess IFN signaling requirements to induce IDO during influenza infection mice lacking intact IFN type I (IFNAR-KO) or type II (IFNγR1-KO) receptor genes were infected with X31 virus and IDO activity was assessed in homogenized lungs and msLNs. Throughout infection IDO activity remained at basal levels in lungs of X31-infected IFNγR1-KO mice (Fig. 4A), relative to basal IDO activity in lungs from uninfected mice (not shown), indicating that IFNγ induced IDO in infected lungs. In contrast, IDO activity was induced to comparable levels in lungs of X31-infected B6 and IFNAR-KO during influenza infection (Fig. 4A), indicating that IFNαβ signaling was not required to induce IDO in infected lungs.

Ablating either IFN type I or type II receptors had no significant effects on IDO activity in msLNs of X31-infected mice up to seven days post-infection (Fig. 4B) when host adaptive immunity clears virus from lungs in this model (Fig. 4C). Since IFN signaling is essential to induce IDO gene transcription these findings suggest that redundant signals from IFN types I or II induce IDO in this period. In contrast, significantly lower IDO activity, relative to WT controls, was observed in msLNs from X31-infected IFNγR1-KO mice following virus clearance (7–9 dpi). Though IDO activity levels were not significantly different (relative to WT mice) in msLNs of X31-infected IFNAR-KO mice, IDO activity levels were significantly higher in IFNAR-KO relative to IFNγR1-KO mice in this period (Fig. 4B), emphasizing that IFNγ is the major IDO inducer in msLNs following virus clearance. Thus, unlike lungs, where IFNγ was essential to stimulate IDO activity during and after infection, redundant IFN type I or type II signals up-regulated IDO activity in msLNs during viral infection.

Virus titers in lungs at the peak of infection (3–6 dpi) were significantly higher (×10^1^–10^2^) in IFNAR-KO mice than in B6 (WT) and IFNγR1-KO mice (Fig. 4C), indicating that IFN type I mediated more effective virus control during X31 infection. However, ablating either IFN type I or type II signaling led to consistent delays in viral clearance (by one day), relative to virus clearance rates in B6 (WT) mice (Fig. 4C). Rates of virus clearance were unaffected by IDO1 gene ablation but virus titers in lungs trended lower (though were not statistically significant) in IDO1-KO mice (×10^1^) relative to WT mice (Fig. 4C), suggesting that IDO may impede host control during X31 infection.

### IDO restrains host CD8 T cell responses to influenza infection

**Figure 5:**
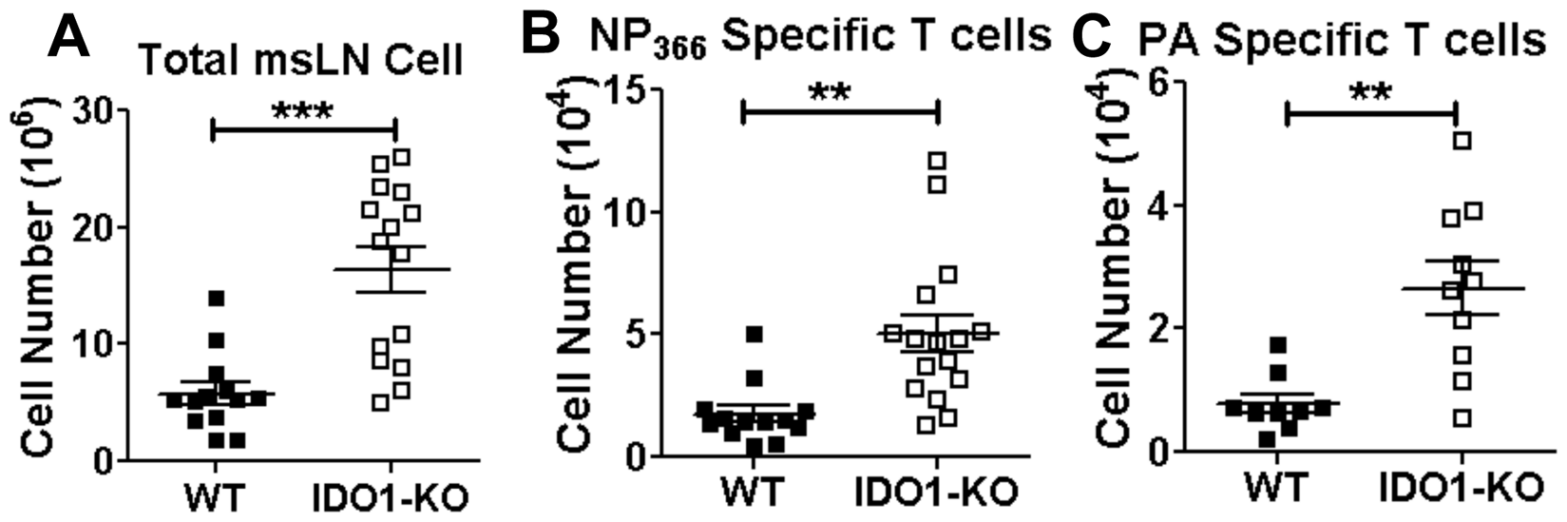
IDO attenuates influenza-specific CD8 T cell responses in X31-infected mice. **A–C.** B6 and IDO1-KO (B6) mice were infected with X31 virus and total numbers of cells and virus-specific CD8 T cell numbers were evaluated in msLNs at 10 dpi using tetramers specific for NP_366_ (B) and PA (C). Data were compiled from 5 experiments, and statistical analyses were performed using Student's unpaired *t* test; **p<0.01, ***p<0.001.

To evaluate if IDO1 gene ablation affected host T cell responses to influenza infection we assessed virus-specific CD8 T cell responses in lungs and msLNs of B6 and IDO1-KO mice by flow cytometric analyses. Total CD8 T cells specific for epitopes derived from NP_366_, PA and were comparable in BAL from X31-infected B6 and IDO1-KO mice (Fig. S1). However msLNs in infected IDO1-KO mice were consistently more inflamed, and total cell numbers in msLNs were significantly higher (∼3-fold) than in B6 mice (Fig. 5A). Moreover, numbers of CD8 T cells specific for NP_366_ and PA influenza epitopes were significantly higher in msLNs (∼2-fold) of IDO1-KO than B6 mice at 10 dpi, indicating that IDO activity restrains clonal expansion of CD8 effector T cells in msLNs. These disparate outcomes for X31-infected WT and IDO1-KO mice were remarkably similar to data reported for PR8-infected mice, even though X31 infection had almost no effect on host morbidity.

### IDO shapes influenza-specific memory CD8 T cell generation

**Figure 6:**
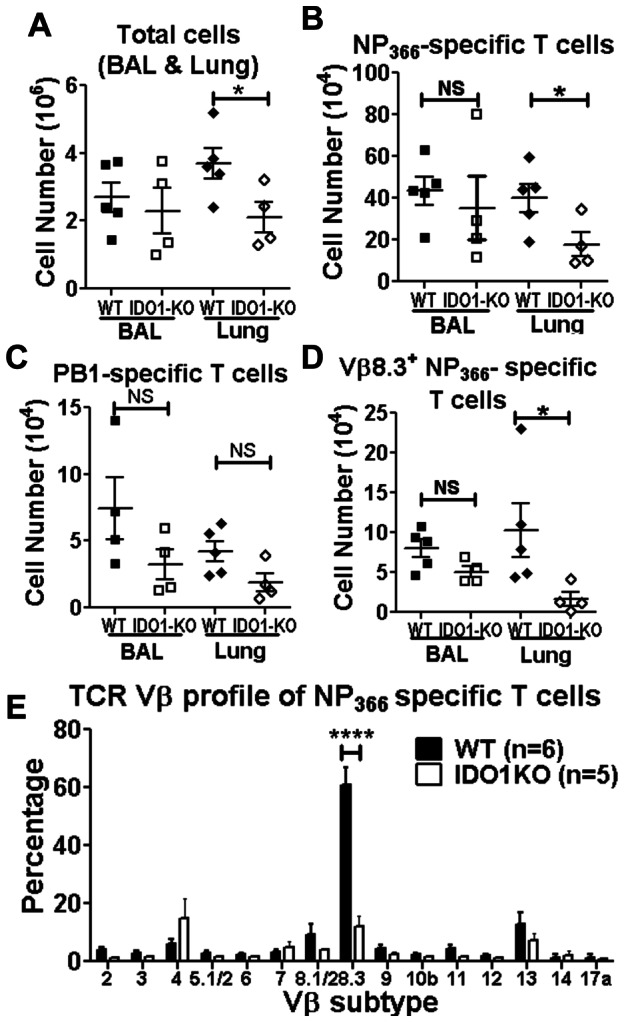
IDO shapes influenza-specific memory CD8 T cell generation. X31-primed WT and IDO1-KO (B6) mice were challenged with PR8 and influenza-specific T cell responses in BAL and lung parenchyma were assessed at 5 dpi. **A–C.** Numbers of total cells (A), NP_366_-specific T cells (B), PB1-specific T cells (C). **D.** Repertoires of NP_366_-specific T cells in BAL and lung parenchyma were analyzed by staining cells for TCR Vβ8.3 at 5 dpi. **E.** Repertoires of long-term NP_366_-specific memory T cells in spleen were analyzed using a panel of antibodies against various TCR Vβ subtypes. Data were compiled from 2 experiments and statistical analyses were performed using Student's unpaired *t* test. *p<0.05, ****p<0.0001.

To assess if IDO1 gene ablation influenced the generation of memory CD8 T cells that protect mice from subsequent challenge with lethal PR8 infection, B6 and IDO1-KO mice were primed with X31 virus then challenged with a lethal dose of PR8 virus (100×mLD50) >30 days after primary infection. CD8 T cell responses to PR8 infection in BAL and lung parenchyma were assessed by flow cytometry. Prior infection with X31 virus protected B6 and IDO1-KO mice from lethal PR8 infection and as expected, sterile viral (PR8) clearance occurred faster (5 dpi) in both mouse strains (data not shown). Numbers of total cells, NP_366_-specific, and PB1-specific CD8^+^ T cells in BAL were comparable between B6 and IDO1-KO mice (Fig. 6A–C), but fewer total cells and NP_366_-specific and PB1-specific CD8^+^ T cells were found in lung parenchyma from PR8-infected IDO1-KO mice (∼2-fold less, Figs. 6A–C). Numbers of PA-specific CD8^+^ T cells in BAL and lung parenchyma were comparable between B6 and IDO1-KO mice (data not shown). Thus, the exaggerated primary NP_366_-specific CD8 T cell responses observed in IDO1-KO mice infected with PR8 (Fig. 2) or X31 (Fig. 5) did not drive exaggerated responses in X31-primed IDO1-KO mice challenged with PR8.

In B6 mice long term NP_366_-specific memory CD8^+^ T cells exhibit dominant usage of Vβ8.3 T cell receptor (TCR) chains [29]. This hallmark feature was not evident when NP-specific CD8 T cells from IDO1-KO mice were analyzed (Fig. 6D). Proportions of Vβ8.3^+^ cells amongst memory CD8 NP_366_-specific T cells from lung parenchyma were significantly lower in PR8-infected IDO1-KO mice than in B6 mice (∼4-fold) as evidenced by overall lower absolute numbers (Fig. 6D) and decreased proportions (Fig. S2) of Vβ8.3^+^ T cells in the pool of NP_366_-specific CD8 T cells present in lung parenchyma, though the proportions of Vβ8.3^+^ CD8 NP_366_-specific T cells in BAL from B6 and IDO1-KO mice were comparable (Fig. 6D). Analyses of TCR Vβ chain usage in long-term memory CD8 NP_366_-specific splenic T cells 30 days after PR8 challenge confirmed these findings (Fig. 6E) as the proportions of gated NP_366_-specific memory CD8 T cells expressing Vβ8.3 were significantly lower in spleens of immune IDO1-KO mice (∼10% Vβ8.3^+^) relative to equivalent T cells from immune B6 mice (∼60% Vβ8.3^+^), which was comparable with previous reports [29], [30]. This prominent repertoire shift away from Vβ8.3^+^ T cells in spleens of IDO1-KO mice did not lead to quantitative differences in NP_366_-specific T cells (data not shown). However, Vβ TCR chain usage was comparable in splenic CD8 T cells from IDO1-KO and B6 mice during primary infection with X31 (Fig. S2), indicating that altered TCR Vβ repertoires were not a consequence of developmental differences in T cell repertoires in IDO1-KO and B6 mice and arose due to adaptive responses to X31/PR8 infection. Loss of dominant Vβ8.3^+^ NP-specific memory CD8 T cells did not lead to compensatory increases in other Vβ TCR isotypes detected by the panel of anti-Vβ TCR mAbs, suggesting that novel Vβ TCR sub-types emerged during recall responses, or that Vβ8.3 dominant memory T cells failed to expand.

### IDO attenuates lung T_H_17 responses during recall responses to influenza infection

**Figure 7:**
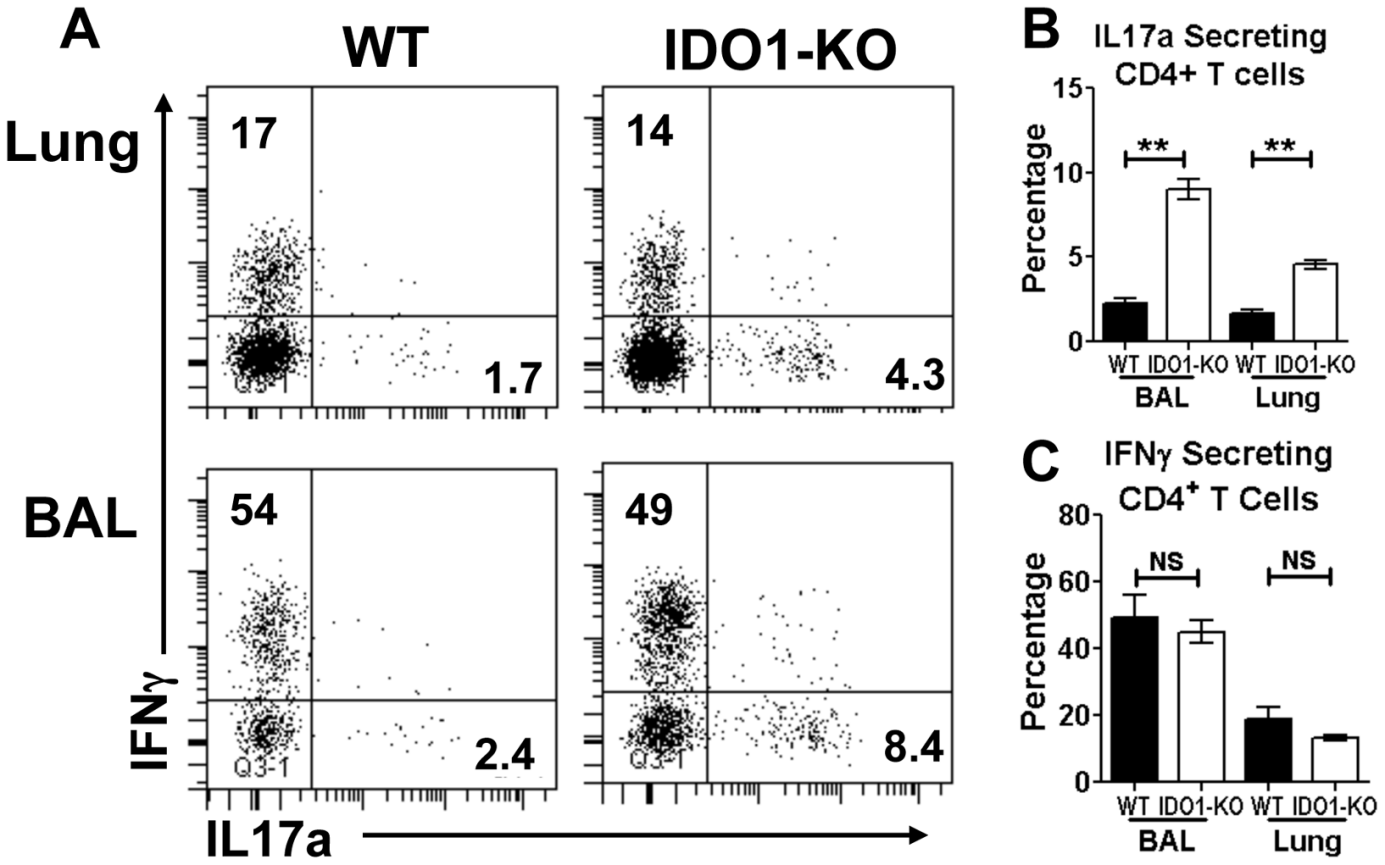
IDO attenuates T_H_17 responses to PR8 challenge. X31 primed WT and IDO1-KO (B6) mice were challenged with PR8 and CD4 T cell status were assessed by FACS analysis of gated CD4 T cells in BAL and lung parenchyma at 5 dpi after *ex vivo* stimulation with PMA/ionomycin and staining to detect intracellular cytokines. **A.** Representative FACS plots for intracellular staining of IL17a and IFNγ in gated CD4 T cells. **BC.** Percentages of IL17a (B) and IFNγ^+^ (C) CD4 T cells are shown. Data were generated from mice primed from various times but re-challenged in the same experiment (2–3/group). Data were analyzed using Student's unpaired *t* test; **p<0.01, NS: non significant.

Lung IDO activity attenuates effector T_H_17 T cell responses that drive lethal pathology during mycobacterial tuberculosis and fungal aspergillosis [31], [32]. To assess if IDO activity also regulated T_H_17 responses X31-infected B6 and IDO1-KO mice were challenged with PR8 and expression of IFNγ and IL17a by CD4 T cells in BAL and lung parenchymal cells from PR8-infected mice was assessed after re-stimulation *ex vivo* (5 hr.+Brefeldin). Proportions of CD4 T cells expressing IL17a were significantly higher in BAL and lungs of IDO1-KO mice relative to B6 mice (Fig. 7A, B). In contrast, proportions of CD4 T cells from PR8-infected B6 and IDO1-KO mice that expressed IFNγ were comparable (Fig. 7A, C). Moreover, enhanced T_H_17 T cell responses did not manifest during primary (sub-lethal) PR8 infection (Fig. S3), indicating that exaggerated T_H_17 T cell responses after PR8 challenge were an indirect consequence of ablating IDO activity during primary (X31) infections.

## Discussion

In this study we show that influenza infection stimulated rapid increase in IDO activity in lungs and lung-associated lymph nodes that persisted long after virus was eliminated from lungs. Ablating IDO1 genes eliminated IDO activity in lungs and associated lymph nodes, and loss of IDO activity led to less morbidity during infection and faster recovery after viral clearance. More robust primary CD8 and recall T_H_17 T cell effector responses also manifested in lungs of infected mice lacking intact IDO1 genes, and loss of IDO function altered the repertoire of virus-specific memory CD8 T cells. Collectively, these findings reveal that induced IDO contributes to increased morbidity and restrains and shapes host T cell responses to influenza infection.

Influenza remains a leading cause of death in the US with >53,000 deaths attributed by the CDC to influenza or influenza-related infections in 2009 [33]. Many patients that die succumb to secondary respiratory infections, such as pneumococcal infections, due to reduced host resistance following primary influenza infections. IDO was implicated as a potential host factor that increases susceptibility to secondary lung infections as mice treated with IDO inhibitor following primary influenza infection exhibited more effective control of subsequent pneumococcal infections, though increased host control of secondary infections did not translate into lower mortality in this co-infection model [6]. Enhanced host susceptibility to secondary bacterial infections manifested rapidly (as soon as 3 dpi) and can persist until long after virus clearance [6], [34]. In the present study, we found that IDO ablation led to significant reduction in morbidity during influenza infections, and to faster recovery after infections were cleared. These findings support the hypothesis that elevated lung IDO activity lowers resistance to opportunistic secondary infections that enhance morbidity, slow recovery, and increase the risk of mortality. IDO activity also suppressed lung Th17 responses, suggesting that IDO may attenuate collateral lung pathology mediated by IL-17, as occurred in a model of pulmonary aspergillosis [31]. Thus, IDO may alleviate cumulative lung pathology caused by recurrent exposure to influenza in humans. Sustained kynurenine release may also lower blood pressure and drive ‘sickness induced depression’ syndromes that modify feeding behaviors to confer potential survival advantages [35], [36].

Of the two homologous genes that encode IDO enzymes in mice [21], only the IDO1 gene encoded IDO activity induced by influenza infection, and intact IDO2 genes did not compensate for loss of IDO1 function in this setting. Similar to the study by Yoshida and colleagues [5] infection with influenza PR8 and the less virulent X31 strain stimulated rapid and substantial increase in IDO activity in lungs that peaked about one week after initial infection and persisted long after sterile viral clearance was achieved. IDO expression was induced in lung epithelial cells from early stages of infection (3 dpi) and was coincident with virus infection. At such early stages of infection no virus-specific lymphocytes were present in lungs to produce IFNγ, the obligate IDO inducer in lung stromal cells, suggesting that innate immune cells such as macrophages and NK T cells may release IFNγ in response to initial influenza infection. Persistence of IDO activity in the absence of direct viral stimulation at later stages after virus is cleared from lungs may reflect continued IFN production or the stability of IDO enzymes during the post-infection recovery phase. Influenza infection stimulated IFN type I (IFNαβ) release to comparable levels in lungs of mice (data not shown) that possessed or lacked intact IFNγ receptor genes, but IFNαβ did not induce lung stromal cells to express IDO, suggesting that these cells are unresponsive to signals from IFNαβ. In contrast, signals from IFNαβ or IFNγ induced IDO in hematopoietic cells in lung-associated lymph nodes, suggesting that dendritic cells that express IDO in response to signals from either IFNαβ or IFNγ [37] may account for IDO activity induced in these settings. Thus distinct cell types in lungs or lung-associated lymph nodes expressed IDO in response to IFN type I or type II signaling following influenza infection.

IDO is induced by many microbial infections but has diametric roles in both host defense and regulating host immunity [38]. IDO mediates innate host defense to some pathogens by lowering the viability of pathogen-infected cells and increasing resistance of neighboring (uninfected) cells to pathogen infection [39], [40]. If IDO mediates host defense to influenza such functions were dispensable, as IDO ablation did not compromise overall host control of influenza infections. IDO also regulated host innate and adaptive immunity in some mouse chronic respiratory infections, such as tuberculosis and aspergillosis, to limit immune-mediated collateral damage to lung tissues during such infections [31], [32]. Moreover, the CD1d ligand alpha-galactosylceramide (αGalCer) used as a natural killer (NK) T cell adjuvant to boost vaccine responses had the paradoxical effect of reducing primary CD8 T cell responses due to IDO induction, even though αGalCer boosted long-term protective memory to influenza vaccination [41]. In the present study we show that IDO attenuated virus-specific CD8 T cell responses to primary influenza infection, modified the repertoire of virus-specific, memory CD8 T cell generated, and impaired lung T_H_17, but not T_H_1 responses following PR8 challenge. However, the T cell regulatory effects mediated by IDO did not compromise overall host control of virus infection, slow viral clearance or reduce protective immunity to PR8 challenge. Thus, IDO has nuanced effects on host T cell primary responses to influenza infection, but IDO persistence following viral clearance conditions lungs to be more susceptible to secondary infections that enhance morbidity and the risk of mortality. It is unclear what evolutionary benefits may counter the apparent detrimental effects of induced IDO activity during influenza infection but IDO-mediated innate host defense, protection from collateral immune-mediated damage, and altered behaviors are potential reasons why IDO activity is a prominent feature of local host responses to influenza infection.

## Supporting Information

Figure S1 **Flu specific CD8 T cells in BAL after X31 infection.** Numbers of NP_366_-specific (A) and PA-specific (B) T cells in BAL were examined using MHC class I tetramers at various times post infection (n = 3).
(TIFF)

Figure S2 **IDO reduces NP_366_-specific memory CD8 T cells expressing TCR Vβ8.3^+^ in lung mesenchyma.** **A.** Mice were primed with PR8, challenged with X31 and tetramers were used to gate NP_366_-specific CD8 T cells in lung parenchyma and then assess surface TCR Vβ8.3 expression. **B.** Vβ8.3**^+^** NP_366_-specific splenic T cells were detected during X31 primary infection at 8 dpi (B). *p<0.05, NS, not significant.
(TIFF)

Figure S3 **IDO ablation does not affect Th1 or Th17 responses to primary PR8 infection.** **A.** B6 (WT) and IDO1-KO mice were infected with PR8 (200 PFU, i/n) and CD4 T cell status in BAL and lung parenchyma at 8 dpi was assessed after *ex vivo* stimulation with PMA/ionomycin and evaluating intracellular cytokine staining by FACS analysis Representative FACS plots to detect intracellular IFNγ and IL-17a staining in CD4 T cells are shown. **BC.** Proportions of IFNγ (B) and IL-17a (C) producing CD4 T cells in WT and IDO1-KO mice during primary PR8 infection (n = 3); NS, not significant.
(TIFF)
