# Supplementary figures and images for: Induction and Role of Indoleamine 2,3 Dioxygenase in Mouse Models of Influenza A Virus Infection
Source: PLoS One. 2013 Jun 13;8(6):e66546. doi: 10.1371/journal.pone.0066546 (PMC3681773; doi:10.1371/journal.pone.0066546)

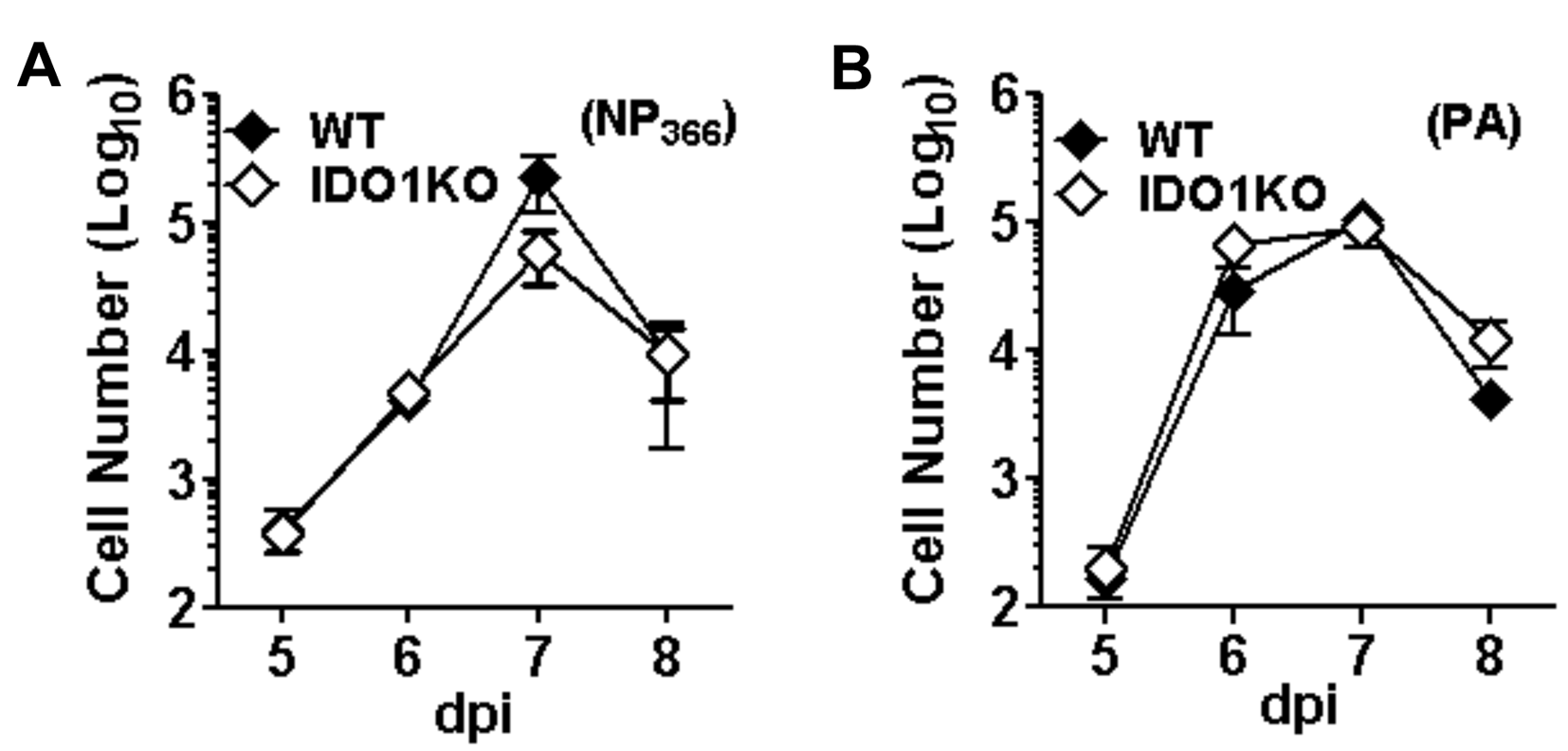

Supplement: Figure S1 — Flu specific CD8 T cells in BAL after X31 infection. Numbers of NP366-specific (A) and PA-specific (B) T cells in BAL were examined using MHC class I tetramers at various times post infection (n = 3). (TIFF) [file pone.0066546.s001.tiff]

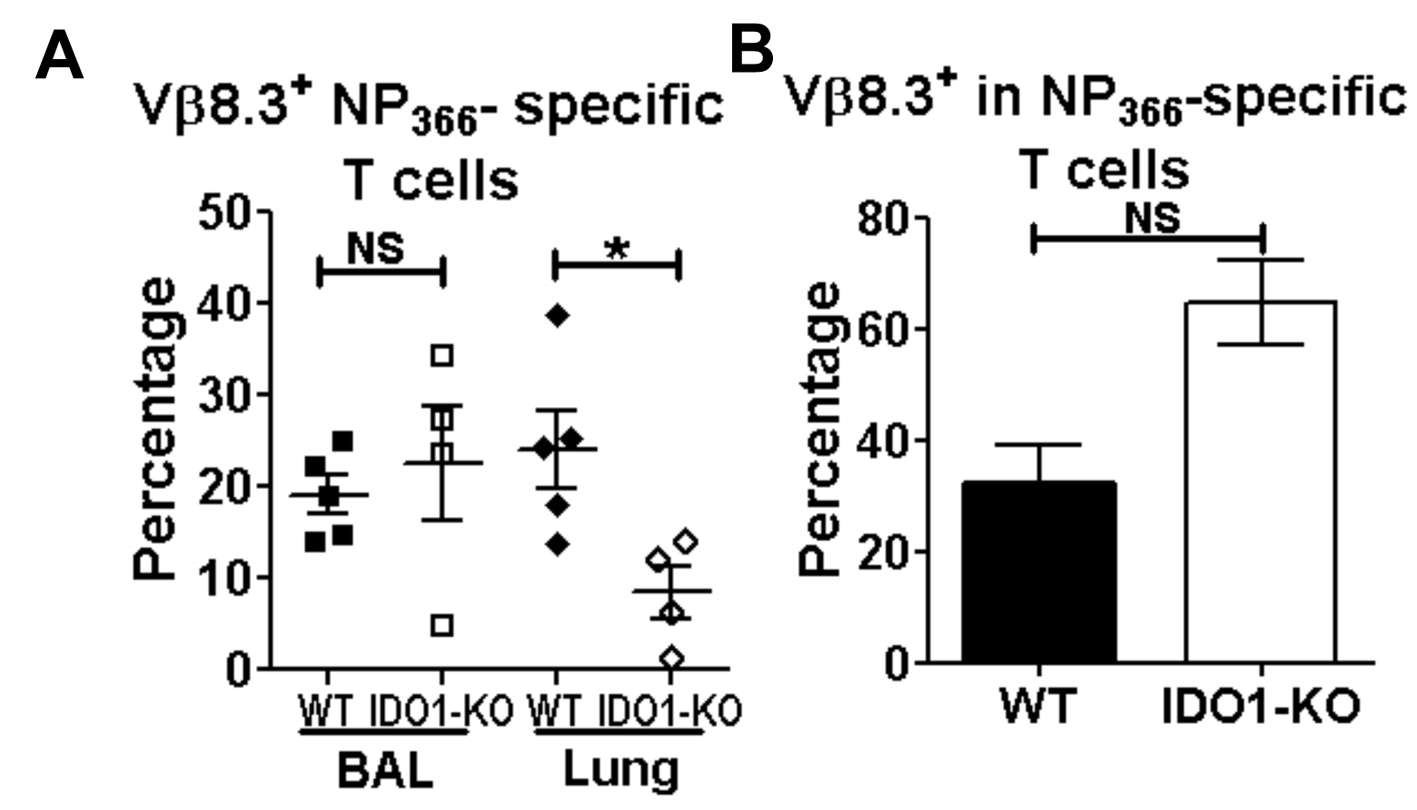

Supplement: Figure S2 — IDO reduces NP366-specific memory CD8 T cells expressing TCR Vβ8.3+ in lung mesenchyma. A. Mice were primed with PR8, challenged with X31 and tetramers were used to gate NP366-specific CD8 T cells in lung parenchyma and then assess surface TCR Vβ8.3 expression. B. Vβ8.3+ NP366-specific splenic T cells were detected during X31 primary infection at 8 dpi (B). *p<0.05, NS, not significant. (TIFF) [file pone.0066546.s002.tiff]

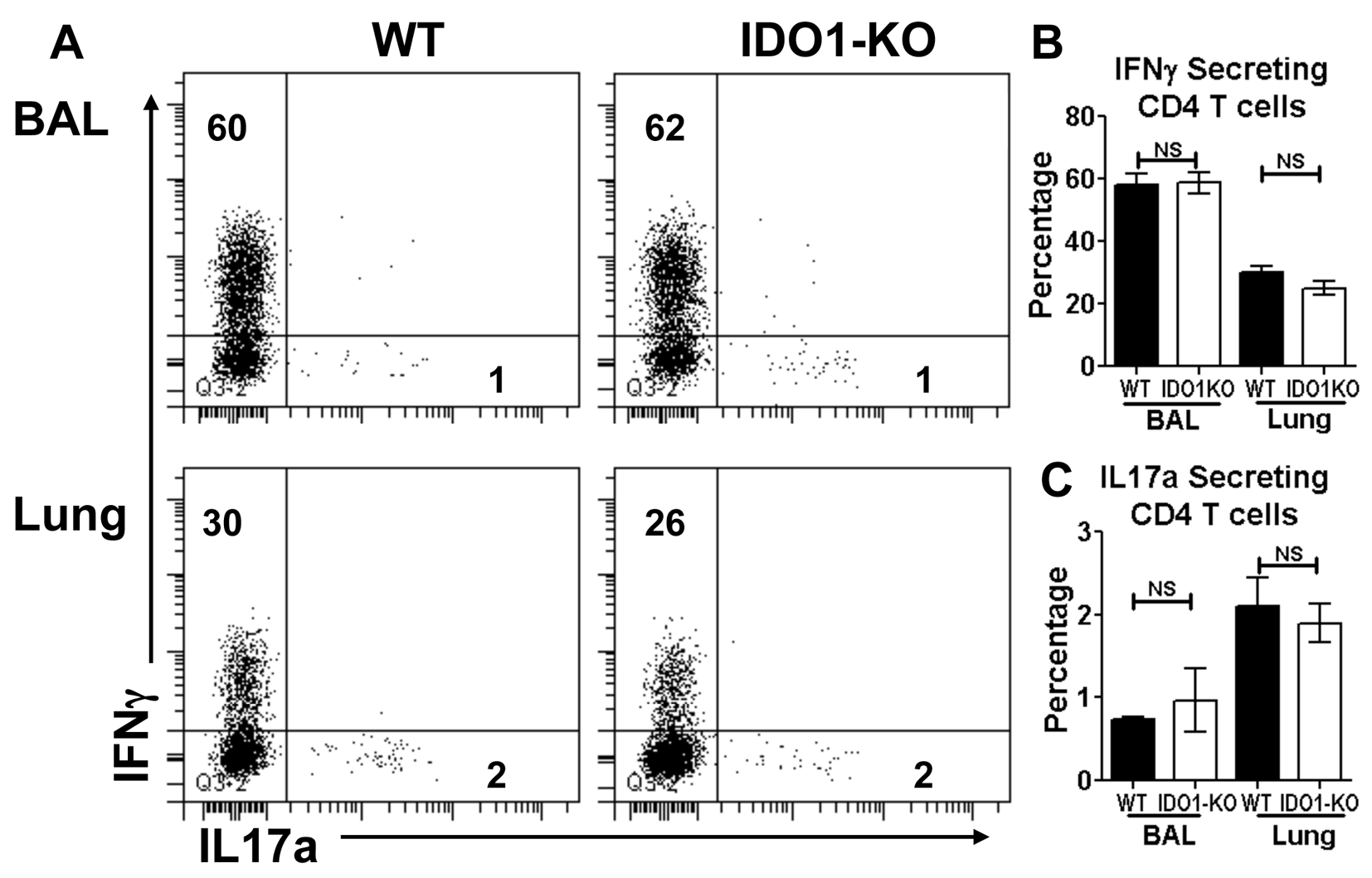

Supplement: Figure S3 — IDO ablation does not affect Th1 or Th17 responses to primary PR8 infection. A. B6 (WT) and IDO1-KO mice were infected with PR8 (200 PFU, i/n) and CD4 T cell status in BAL and lung parenchyma at 8 dpi was assessed after ex vivo stimulation with PMA/ionomycin and evaluating intracellular cytokine staining by FACS analysis Representative FACS plots to detect intracellular IFNγ and IL-17a staining in CD4 T cells are shown. BC. Proportions of IFNγ (B) and IL-17a (C) producing CD4 T cells in WT and IDO1-KO mice during primary PR8 infection (n = 3); NS, not significant. (TIFF) [file pone.0066546.s003.tiff]
